# Infection by cyst nematodes induces rapid remodelling of developing xylem vessels in wheat roots

**DOI:** 10.1038/s41598-020-66080-z

**Published:** 2020-06-03

**Authors:** Kara A. Levin, Matthew R. Tucker, David McK. Bird, Diane E. Mather

**Affiliations:** 1 School of Agriculture, Food and Wine, Waite Research Institute, University of Adelaide, PMB 1, Glen Osmond, 5064 South Australia Australia; 2 Department of Entomology and Plant Pathology, North Carolina State University, Raleigh, North Carolina 27695 USA

**Keywords:** Parasitism, Confocal microscopy

## Abstract

Cyst nematodes induce host-plant root cells to form syncytia from which the nematodes feed. Comprehensive histological investigation of these feeding sites is complicated by their variable shape and their positions deep within root tissue. Using tissue clearing and confocal microscopy, we examined thick (up to 150 μm) sections of wheat roots infected by cereal cyst nematodes (*Heterodera avenae*). This approach provided clear views of feeding sites and surrounding tissues, with resolution sufficient to reveal spatial relationships among nematodes, syncytia and host vascular tissues at the cellular level. Regions of metaxylem vessels near syncytia were found to have deviated from classical developmental patterns. Xylem vessel elements in these regions had failed to elongate but had undergone radial expansion, becoming short and plump rather than long and cylindrical. Further investigation revealed that vessel elements cease to elongate shortly after infection and that they later experience delays in secondary thickening (lignification) of their outer cell walls. Some of these elements were eventually incorporated into syncytial feeding sites. By interfering with a developmental program that normally leads to programmed cell death, *H. avenae* may permit xylem vessel elements to remain alive for later exploitation by the parasite.

## Introduction

As the conduits through which water and mineral nutrients are transported from roots to shoots, the xylem vessels of roots are critically important for plant growth and development. As roots grow, xylem precursor cells differentiate from the root apical meristem and develop into protoxylem and metaxylem vessels^1^. Metaxylem vessel elements elongate and begin to mature. They undergo secondary thickening (lignification) and programmed cell death and the end walls between adjacent elements erode to form hollow tubes^2^. In the seminal roots of wheat (*Triticum aestivum* L.) and other cereal crops, there are multiple peripheral metaxylem (pMX) vessels arranged around one large central metaxylem (cMX) vessel. The pMX matures first and the cMX matures later^3^. In these systems, the cMX can account for up to 80% of water transport^4^.

Many root pathogens and parasites take advantage of xylem tissue to sustain their own nutrient requirements. This can interfere with water transport and lead to symptoms resembling those induced by water stress^5,6^. Further, some sedentary parasitic nematodes are known to influence the development of plant vascular tissue. This has been thoroughly investigated for root knot nematodes (RKN, *Meloidogyne* spp.), which induce the development of new vascular tissue around their ‘giant cell’ feeding sites^7^. In contrast, cyst nematode parasitism has not been reported to affect xylem development.

Like root knot nematodes, cyst nematodes (CN, including *Heterodera* spp. and *Globodera* spp.) are sedentary endoparasites of plants. Juvenile CNs emerge from soil-borne cysts and enter the elongation zones of host plant roots. After migrating intracellularly through the root cortex^8^, they select initial feeding cells, into which they inject complex mixtures of effectors. These effectors modify host cell metabolism, leading to partial breakdown of plant cell walls and merging of cytoplasm between cells^9,10^. The resulting syncytia ‘recruit’ adjacent cells and can eventually incorporate hundreds of cells. They act as transfer cells^11^, delivering nutrients to nematodes.

Cyst nematodes rely upon ongoing nutrient transfer from surrounding cells to feeding sites. It has been proposed that developing syncytia are initially isolated and rely upon transport proteins to obtain nutrients, but later become symplasmically connected to the nutrient-dense phloem^12^. Accumulated solutes are stored within syncytia^13^, allowing intermittent feeding by nematodes. As the syncytia expand, they may come into contact with metaxylem vessels^14^ and incorporate those vessels as part of the syncytia^15–17^. Such expansion may enhance water uptake to support nematode development^18^ and/or help maintain high turgor pressure^19^ within the syncytia.

Much of what is known about CN feeding sites has been learned from microscopic analysis of thin sections of infected root tissue. Optical microscopy has provided detailed views of cells and tissues^20,21^, while electron microscopy has provided detailed views of ultrastructure within cells^11,22,23^. While these approaches provide detailed cytological information regarding feeding site development, each thin section provides only a two-dimensional view of the feeding site and other features. Reconstitution of three-dimensional shapes is possible using such approaches, but to examine the relative positions of nematodes, feeding sites and surrounding tissues would require many serial sections.

Recently, alternative microscopic techniques have been used to generate three-dimensional (3D) models of infected root tissue. The use of tissue clearing reagents such as ClearSee^24^ and TOMEI^25^, combined with confocal microscopy, have made it possible to capture and examine 3D structures within nematode-infected roots without physical sectioning. This is has been achieved for *Arabidopsis thaliana* L. roots infected by the root-knot nematode *Meloidogyne incognita*^25,26^ and for Chinese milkvetch (*Astralagus sinicus* L.) roots infected by soybean cyst nematode (*Heterodera glycines*)^27^. These host species have thin roots that are amenable to wholemount processing, but may not be entirely realistic models for CN-susceptible host plants with thicker roots or different root anatomy.

Laser ablation tomography has been applied to segments of barley roots infected by the cereal cyst nematode (CCN; *Heterodera avenae* W.)^28^. This approach revealed the shapes and relative positions of nematodes, feeding sites and surrounding tissue, but with less detail than can be seen with microscopy.

Here, we report on the development of a systematic approach for preparation and confocal microscopy of thick sections of root tissue and present highly detailed images obtained from the application of this approach to CCN-infected roots of wheat (*Triticum aestivum* L.). Based on these images, we report on the discovery of a striking yet previously unreported modification of metaxylem vessels and present clear evidence of the ‘invasion’ of metaxylem vessels by feeding sites.

## Results

### A systematic approach for preparation and microscopic analysis of root tissue

**Figure 1:**
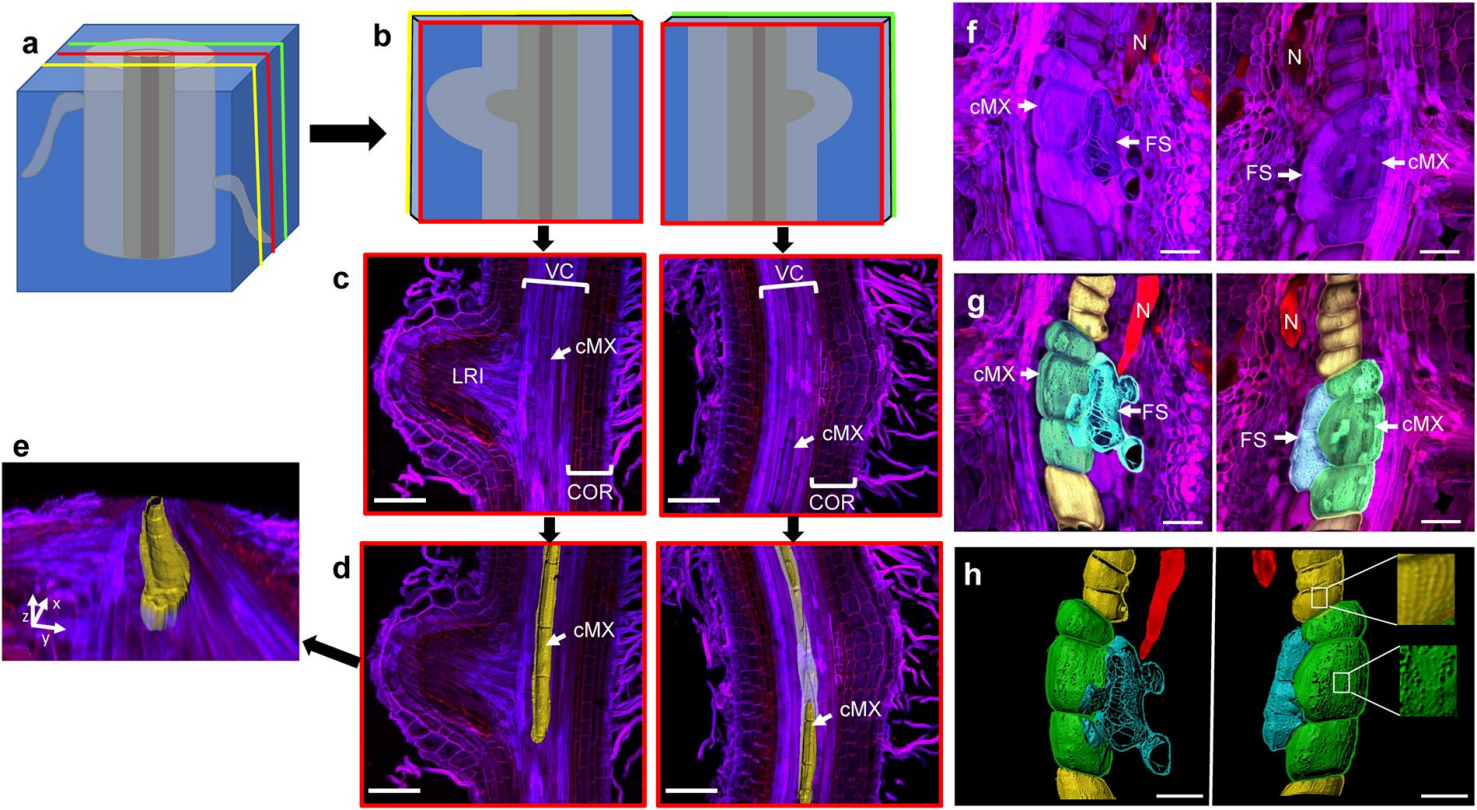
Method to image and model thick sections of control and infected wheat root tissue. (**a**) Root tissue was embedded and cut into 150 µm longitudinal sections. (**b**) Sections containing the central part of the vascular bundle were chosen. (**c**) Sections were stained with calcofluor white and propidium iodide and then imaged using a confocal microscope and processed with Nikon NIS-Elements to produce a maximum projected image. (**d**) Using IMARIS software, the central metaxylem vessel was manually selected and highlighted in order to render and (**e**) rotate a three-dimensional surface model of the central metaxylem. This same process was used to analyse wheat seminal root tissue at 15 d after inoculation with cereal cyst nematodes. (**f**) Maximum projected confocal images stained and showing facing surfaces of two consecutive sections containing a nematode (N), a central metaxylem (cMX) vessel and a feeding site (FS). (**g**) Features manually selected and coloured: the nematode in red, the feeding site in blue and the central metaxylem in green (three vessel elements that have been ‘invaded’ by the feeding site) and yellow. (**h**) Three-dimensional models of the selected features. A detailed rotation of the left-hand model is shown in Supplementary Video S1. (VC) vascular cylinder (cMX) central metaxylem, (LRI) lateral root initial, (COR) cortex. Scale bar (**a–d**) 200 µm (**f–h**) 100 µm.

An approach was developed to examine anatomical features within wheat roots. This involved embedding short segments of wheat seminal roots in agarose, followed by sectioning at 150 µm intervals (Fig. 1a). With examination of the resulting sections by light microscopy, it was possible to select pairs of adjacent sections that contain features of interest (Fig. 1b). The selected sections could be stained and then examined using confocal microscopy. Using data from the facing surfaces of a pair of sections, a Z-stack of approximately 100 µm depth could be obtained. This provided maximum projected confocal images, within which key cell and tissue types could be distinguished, including root hairs, epidermal cells, cortical cells and vascular tissue (Fig. 1c). In some samples, lateral roots or lateral root initials were visible. With IMARIS software, central metaxylem (cMX) vessels were manually selected from images of longitudinal sections (Fig. 1d) to enable rendering of surfaces and generation of 3D models (Fig. 1e). As expected, cMX vessels formed apparently hollow cylindrical tubes, with ‘scars’ marking where individual vessel elements had formerly been separated by end walls.

When the same methods were applied to CCN-infected segments of wheat roots, feeding sites and/or nematodes were visible, often located near the cMX (Fig. 1f). In contrast to the long cylindrical cMX vessel elements observed in non-infected roots, cMX vessel elements near feeding sites were short and plump. Some elements appeared to be separated by intact or nearly intact end walls. Central metaxylem vessels, feeding sites, and nematodes were selected (Fig. 1g) for the development of 3D models. These models clarified the relative positions of feeding sites and xylem vessels (Fig. 1h) and revealed complex networks of remnant inner cell walls within feeding sites (Fig. 1h, Supplementary Video S1). They also revealed pores in outer walls of xylem vessels (Fig. 1h, right panel) and some direct connections between vessel elements and feeding sites (Fig. 1h, left panel).

### Development of metaxylem vessels and feeding sites in nematode-infected roots

**Figure 2:**
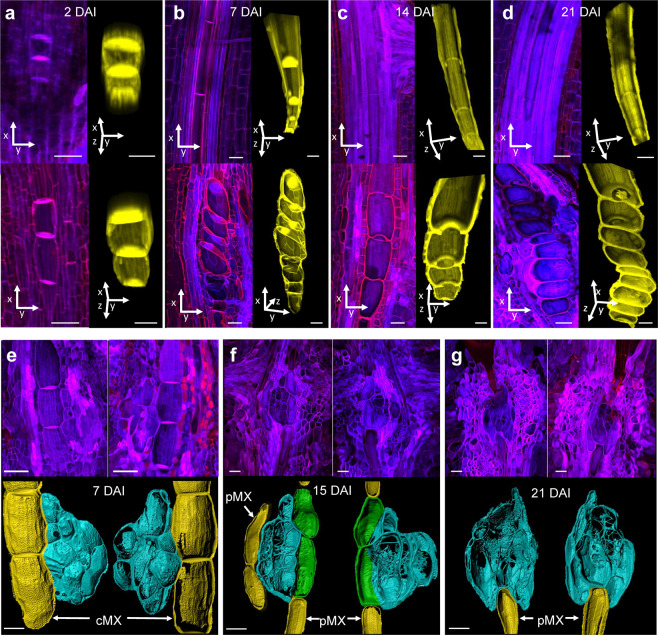
Central metaxylem development in wheat seminal roots with and without infection by cereal cyst nematodes. (**a–d**) The upper panel shows longitudinal sections from control (mock-inoculated) roots and the lower panel shows longitudinal sections of inoculated roots at (**a**) 2 d after inoculation (DAI), (**b**) 7 DAI, (**c**) 15 DAI, and (**d**) 21 DAI. Left-hand panels show maximum projection confocal images (processed with Nikon NIS-Elements) of tissue stained with calcofluor white (blue) and propidium iodide (red). Right-hand panels show the corresponding three-dimensional models (produced using IMARIS) of central metaxylem (cMX) vessels, coloured yellow and tilted to show vessel end walls. (**e,f**) Development of cereal cyst nematode feeding sites and xylem connectivity in wheat seminal roots. (**e**) A feeding site and a cMX vessel at 7 d after inoculation (DAI), (**f**) a feeding site and two peripheral metaxylem (pMX) vessels at 15 DAI and (**g**) a feeding site and a pMX vessel at 21 DAI. Each upper panel shows maximum projection confocal images (processed with Nikon NIS-Elements) of the facing surfaces of two consecutive sections of tissue stained with calcofluor white (blue) and propidium iodide (red). Each lower panel shows isolated three-dimensional models (produced using IMARIS) of feeding sites in aqua and xylem vessels in green (vessel elements that have been ‘invaded’ by the feeding site) and yellow. Rotation of the models is shown in Supplementary Video S2. Scale bar 50 µm.

To investigate the effects of CCN infection on cMX development over time, inoculated roots were examined and compared to mock-inoculated (control) roots at 2, 7, 15, and 21 d after inoculation (DAI). At 2 DAI, cMX vessel elements in infected roots were similar in length, but slightly plumper, than those in control roots (Fig. 2a). By 7 DAI, the cMX vessel elements in control roots were fully elongated while those in infected roots remained short and had expanded radially (Fig. 2b). End walls between vessel elements were still present but appeared stretched and thin in infected roots (Fig. 2b, lower panel). By 15 DAI, the end walls between vessel elements in control roots had broken down, leaving only thin remnants (Fig. 2c, upper panel). In infected roots, some end walls had broken down, leaving thick ridges (Fig. 2c, lower panel), while other end walls persisted. Even at 21 DAI, some fully intact end walls were still visible between very short and wide vessel elements (Fig. 2d, lower panel).

In infected regions sampled at 7, 15, and 21 DAI (at least four samples from each time point) feeding sites were always directly adjacent to or connected with metaxylem vessels. For example, a feeding site in tissue sampled at 7 DAI (Fig. 2e; Supplementary Video S2a) was directly adjacent to the cMX but separated from it by intact cell walls. A feeding site in tissue sampled at 15 DAI (Fig. 2f; Supplementary Video S2b) was located between two pMX vessels. In one of these vessels, two elements immediately adjacent to the feeding site were plump, had apparently porous cell walls and had direct connections with the feeding site. A feeding site sampled at 21 DAI (Fig. 2g; Supplementary Video S2c) appeared to have entirely overtaken a section of a pMX vessel.

### Secondary thickening of cell walls in nematode-infected roots

**Figure 3:**
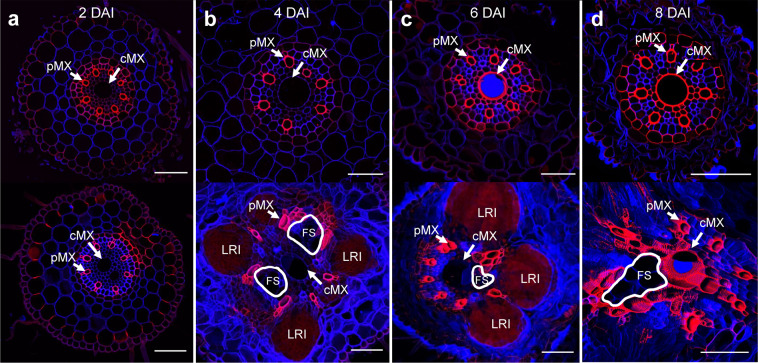
Transverse sections of wheat seminal roots treated with a lignin-specific stain. The upper panels show sections of control (mock-inoculated) roots. The lower panels) show sections of roots inoculated with cereal cyst nematodes. All sections were stained with basic fuchsin (red) and calcofluor white (blue), imaged using a confocal microscope and processed with Nikon NIS-Elements to produce a maximum projected images. (**a**) 2 d after inoculation (DAI), (**b**) 4 DAI, (**c**) 6 DAI (**d**) 8 DAI. (LRI) lateral root, (FS, circled in white) feeding site, (pMX) peripheral metaxylem, (cMX) central metaxylem. Scale bars: 100 µm.

As xylem elements mature, their outer cell walls undergo secondary thickening, a process that involves lignification. When transverse sections of infected and control roots were treated with the lignin-specific stain basic fuchsin, lignin was not detected in the cell walls of feeding sites, but there were differences in the lignification of surrounding cells. At 2 DAI (Fig. 3a), lignin was detected in the cell walls of protoxylem, pMX and exodermis in both control and infected roots. In infected roots, lignin was also detected in the walls of some groups of endodermal and cortical cells. At 4 DAI and later (Fig. 3b–d), the 3D structure of some features (especially the pMX) was more obvious in infected roots than in control roots, probably because of curvature of infected regions of roots. In infected roots, lignin was detected in the walls of some cells adjacent to the pMX. Over time, it became increasingly difficult to distinguish the pMX of infected roots from surrounding cells. At 6 DAI (Fig. 3c), control roots exhibited considerable lignification of the outer walls of the cMX and some lignification of endodermal cell walls. In infected roots, lignification of the outer walls of the cMX was not observed until 8 DAI (Fig. 3d). Intact end walls were sometimes visible within cMX vessels in infected roots (Fig. 3d), and only the outer rims of these walls were lignified.

## Discussion

Motivated by an interest in examining the structure and positions of cyst nematode feeding sites within thick roots of crop plants, we established a systematic approach to prepare and observe root tissue sampled from the seminal roots of wheat seedlings. Key elements of this approach include application of an optical clearing agent, tissue sectioning at 150 μm intervals, selection of adjacent sections containing features of interest, application of confocal microscopy and generation of 3D models. In combination, these methods enabled high-quality examination of feeding sites and provided new information on spatial relations of features within cereal roots. Use of a clearing agent (ClearSee) allowed deep penetration of confocal lasers into the thick sections. Imaging of facing surfaces of adjacent tissue sections provided an ‘open-book’ view of complex structures. Finally, 3D modelling made it possible to isolate specific structures and rotate them in space.

When applied to CCN-infected roots, this approach provided detailed 3D models of feeding sites and metaxylem vessels. These models confirmed the irregular shapes of feeding sites and provided detailed views of intricate cell wall networks within those sites. Some metaxylem vessel elements in infected roots differed markedly from those in the roots of mock-inoculated control plants, with sections of cMX vessels near CCN feeding sites consisting of series of short, plump, bubble-like elements. The end walls between these elements did not erode as early or as completely as their counterparts in uninfected roots. Further, end walls that did erode left thick ridges within xylem vessels, rather than the subtle ‘scars’ that marked the former positions of vessel element end walls in uninfected roots. None of these features have been reported previously.

Examination of tissue samples taken at various times after inoculation revealed that structural modification of the cMX begins soon after infection, certainly by 7 DAI (when all feeding sites would have been initiated^15^) and possibly as early as 2 DAI (when nematodes would be within the root, but not all would have selected an initial feeding cell). Instead of elongating and becoming cylindrical, cMX vessel elements near infection sites expanded radially. Relative to their counterparts from uninfected roots, affected xylem vessel elements underwent secondary thickening (lignification) somewhat later and tended to retain their end walls longer. Further, the outer rims of their end walls underwent secondary thickening, leading to the thick ridges mentioned above. Overall, nematode infection appears to interfere with cell elongation (and/or favour radial growth), causing the formation of bubble-like vessel elements.

Considering that microscopy has been extensively used over many decades to examine CN feeding sites^22,29–31^, it is surprising that nematode-induced bubble-like xylem elements have not previously been reported. One possible explanation is that microscopy has been mainly applied to transverse sections, in which xylem modification would not be obvious. Even when longitudinal sections were used, research interest was focused on nematodes and/or their feeding sites, and structural modification of the xylem might have been missed. Consistent with this interpretation, some published micrographs (Fig. 2c and Fig. 4e of Seah *et al*.^20^ Fig. 2e of Aditya *et al*.^21^) include views of cells that resemble the bubble-like vessel elements reported here, but were not labelled as xylem tissue. Application of light or electron microscopy to sectioned tissue provides information only for a single plane through the tissue, making it difficult to appreciate the full complexity of 3D structures. Confocal microscopy has provided 3D views of whole-mount CN-infected root tissue for Arabidopsis^25,26^ and Chinese milkvetch^27^, but these plants have thinner roots (approximately 200 µm in infected regions) than the wheat plants used here (between 600 and 800 µm in infected regions). Further, the vascular anatomy of the seminal roots of wheat and other cereals is quite different from that of Arabidopsis roots, which lack a prominent cMX. The combination of optical clearing, confocal microscopy, 3D modelling and the use of seminal roots of wheat made it possible to discover a previously unreported phenomenon.

**Figure 4:**
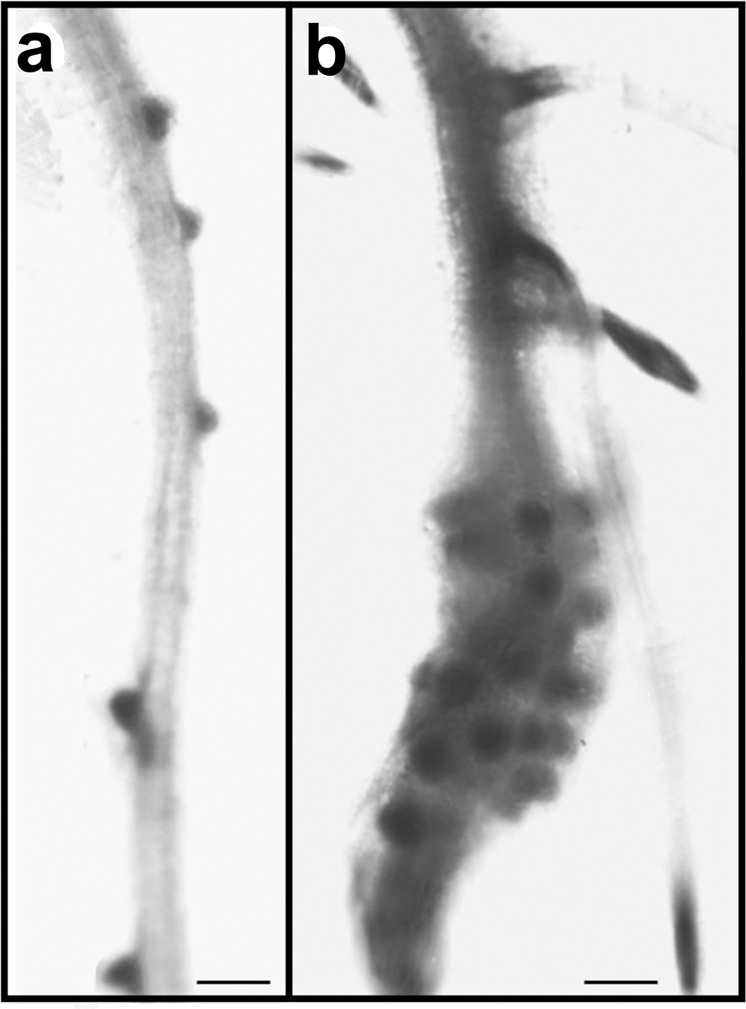
Proliferation of lateral root formation on wheat seminal roots infected with cereal cyst nematodes. (**a**) A control (mock-inoculated) root, with five emerging lateral roots. (**b**) A root at 7 d after inoculation with cereal cyst nematodes, with three advanced lateral roots above the infection site and many lateral roots emerging at the infection site. Scale Bar: 500 µm.

Most of the bubble-like xylem elements examined here were in cMX vessels. These vessels are very large, making any modification of their structure quite obvious. Modification of pMX vessel elements was sometimes observed (e.g. Figure 2f) but tended not to be as extreme as in cMX vessel elements. This is consistent with the fact that pMX (also known as early-maturing metaxylem) matures much earlier than cMX (late-maturing metaxylem). This maturity difference was evident in samples stained with basic fuchsin. Even at 2 DAI, the pMX vessel walls were already highly lignified. Compared to the immature and not yet lignified cMX, pMX may be better able to resist the effects of nematode infection.

The success of developing CN feeding sites relies on access to water and nutrients from the plant’s vascular tissue^31^, including the xylem. It has previously been reported that xylem contact is important for syncytial development^15,16,23,32^, that solute flow from xylem vessels is a limiting factor for syncytial efficiency^22^ and that contact and connection with xylem vessels is important for the development of syncytia^14^. The results presented here demonstrate the structure and extent of the relationship between feeding sites and host plant xylem vessels. Our 3D models confirmed that feeding sites are initiated near metaxylem vessels and maintain close contact with those vessels throughout development. Some feeding sites ultimately ‘invade’ vessel elements, making it difficult to distinguish vessel elements from feeding sites. The cell walls of metaxylem vessel elements that were (or may be destined to be) connected with feeding sites appeared porous, resembling the walls of phloem sieve cells, which serve as major conduits for transport of photosynthates.

Overall, it appears that infection of wheat roots by cereal cyst nematodes triggers a reprogramming of nearby xylem vessel elements, preparing them to be suitable for connection with, or integration into, feeding sites. Cell elongation is impeded (and/or radial expansion is promoted) and cell maturation is delayed. Nematode-induced xylem modification may be an important element of the parasitic mechanism of sedentary nematodes. This is consistent with ideas discussed by Bird and Wilson^33^ and elaborated into a model by Bird^34^, who pointed out similarities between nematode feeding sites (CN syncytia and RKN giant cells) and developing xylem.

The phenomenon reported here differs from previously reported effects of parasitic nematodes on vascular tissue. It is well known that networks of *de novo* xylem and phloem cells develop around RKN feeding sites (giant cells)^7^. In contrast, the response reported here for CCN involves cells that have already differentiated into cMX or pMX vessel elements but fail to elongate and are delayed in (or prevented from) undergoing programmed cell death. While vascular tissue proliferation in response to RKN infection could be a plant response that helps maintain nutrient and water transport for the host plant, remodelling of vascular tissue in response to CCN infection may benefit the parasite more than the host.

Given the importance of the cMX for water transport^4,35^, delayed maturation of cMX vessel elements and retention of end walls between cMX vessel elements could significantly impede water transport within roots. This raises the question of how the host plant is able to survive. One possible explanation lies in the well-known proliferation of lateral roots near feeding sites (e.g., Fig. 4). These new roots may increase overall water uptake and ensure adequate water supply to xylem vessels above feeding sites and to the feeding sites themselves. Further, it is possible that new xylem vessels differentiate within the stele. This might explain the additional lignified cells that were observed near pMX and feeding sites as early as 4 DAI.

The systematic approach that was developed here for the examination of vascular tissue in wheat seminal roots infected by CCN could be applied to other plant species and other research questions. The 3D analysis of CCN feeding sites clearly revealed the structure of feeding sites and their connections with xylem vessels, while also enabling the discovery of previously unreported effects of nematode infection on xylem vessel anatomy. These observations lead to intriguing questions on how xylem remodelling is induced, on whether and how it benefits the parasite and/or affects the host plant.

## Materials and Methods

### Nematodes, plant material, growth conditions, and inoculation

Cereal cyst nematode (*Heterodera avenae* Woll., pathotype Ha 13) inoculum was prepared as previously described^21^. Cysts (originating from soil collected at Winulta and South Kilkerran on the Yorke Peninsula, South Australia) were mixed with organic material and placed in synthetic silk bags to be used as nematode ‘farms’. These nematode farms were incubated in water at 5 °C in darkness. One day prior to inoculum preparation, the farms were rinsed to remove nematodes that had already hatched. On the day of inoculum preparation, water from the nematode farms was washed through a 38 µm sieve to collect freshly hatched J2 nematodes. Nematode numbers were counted for 20 µL samples and the concentration of inoculum was adjusted to approximately 2000 nematodes per mL.

**Figure 5:**
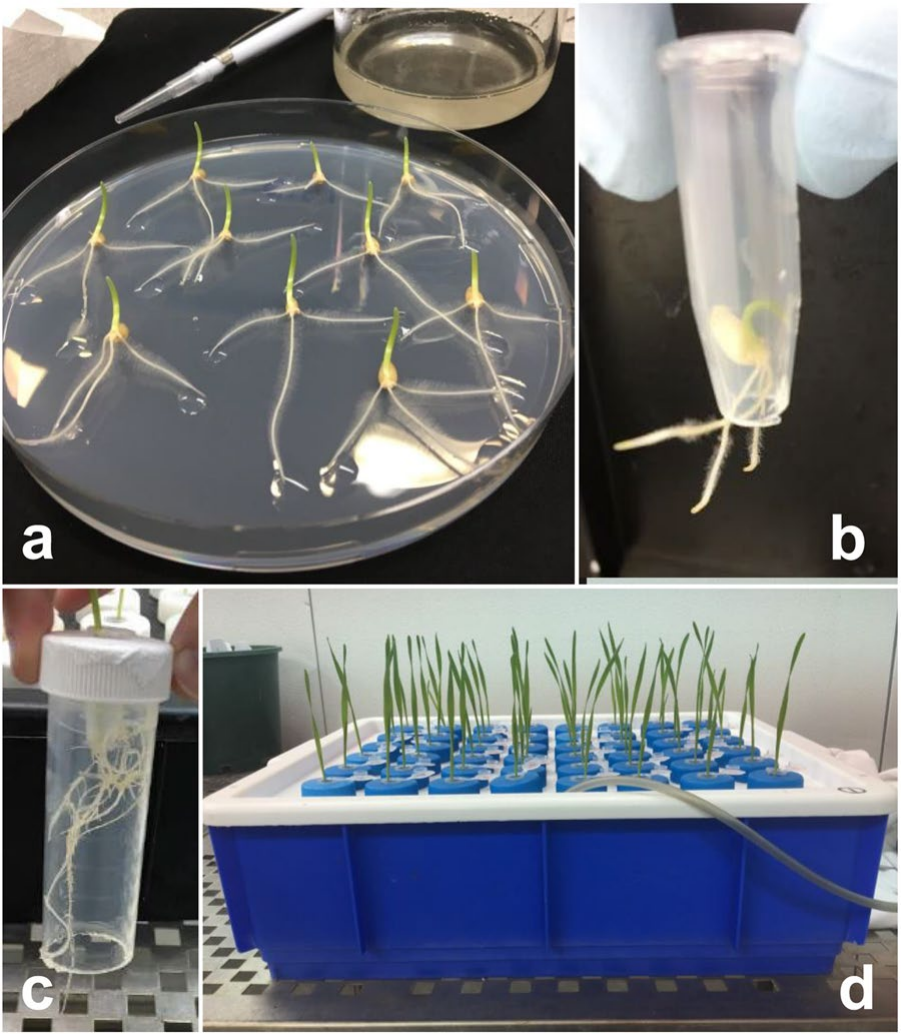
Experimental setup and growth hydroponics system. (**a**) Seedlings germinated and inoculated on agar plates. (**b**) Seedling transferred to Eppendorf tube with tip removed. (**c**) Tube placed into a 50 mL tube with base removed. (**d**) Tubes arranged in an aerated hydroponic tank containing nutrient solution.

Seeds of the wheat (*T. aestivum*) line TMDH82 (a CCN-susceptible doubled haploid line derived from the F_1_ generation of a cross between the Australian wheat cultivars Trident and Molineux) were surface sterilised in 3% sodium hypochlorite (NaOCl) for 10 min on a shaker, then rinsed three times with sterilised water for 5 min. Seeds were placed on sterile 2% agar plates and kept in darkness at 5 °C for 24 h. The agar plates were then placed in a controlled environment chamber at 15 °C with a 12 h: 12 h, light: dark cycle. After 3 d, when seeds had germinated and seedling seminal roots were 2–3 cm long, each seminal root tip was inoculated with 20 µL inoculum or (for control plants) sterile water. Once inoculated, seedlings were kept on agar for 24 h (Fig. 5a). Each seedling was then washed in water, then gently pushed into a 1.5 mL Eppendorf tube from which the tip had been cut off (Fig. 5b). That tube was then placed into a 50 mL tube from which the base had been cut off (Fig. 5c). The 50 mL tube was then placed into a hydroponic tank (Fig. 5d) containing modified Johnson’s solution^36,37^ with constant aeration. The solution was changed twice a week.

### Clearing and sample preparation of root tissue for confocal microscopy

At various times after inoculation, wheat plants were collected and short segments (2–4 mm) were excised from the seminal roots. For inoculated plants, visibly swollen regions were excised. For mock-inoculated control plants, corresponding regions were excised from roots at the same distance from base of the root. Each piece of harvested tissue was placed into an Eppendorf tube, covered with ClearSee solution and incubated at room temperature for 30 d. ClearSee solution (prepared as described^24^) was changed weekly. After 30 d, tissue samples were washed with 0.01 M phosphate-buffer saline (1X PBS) for 1 min, then washed in sterile water for 1 min. Tissue samples were embedded in 4% agarose and sectioned longitudinally at 150 µm intervals using a Leica VT1200 vibratome (Leica Biosystems, Nussloch, Germany). Resulting sections were screened by light microscopy (Nikon Eclipse Ci-L, Tokyo, Japan) and pairs of adjacent sections containing vascular tissue were selected. The selected sections were incubated overnight in ClearSee solution at room temperature. Sections were rinsed twice with 1X PBS and once with sterile water, then stained with a 1:1 mix of 0.1% calcofluor white in 20% EtOH and 10 µg mL^−1^ propidium iodide in water for 20 min. Sections were washed three times with water and mounted in wells of three-well Teflon microscope slides. A drop of 50% glycerol was added to each section and a cover slip was applied.

### Sample preparation for examination of feeding site development

Inoculated plants were collected at 7, 15, and 21 d after inoculation (DAI). Visibly swollen regions were excised from their roots. Tissue was cleared, embedded, and sectioned as described above. Resulting sections were screened by light microscopy and sections containing the feeding site were selected. For each sampling time, at least three pairs of adjacent sections were examined, each from a different root.

### Sample preparation for examination of xylem development

Three inoculated plants and three control plants were collected at each of 2, 7, 15, and 21 DAI. For each inoculated plant, the distance from the base of the root was measured for each swollen region. Swollen regions, and the corresponding regions on control roots, were excised. Tissue samples were immediately embedded in 4% agarose without clearing. Longitudinal sections were cut at 150 µm intervals and screened by light microscopy. Sections containing central metaxylem were selected and immediately stained with 0.1% calcofluor white in 20% EtOH and 10 µg mL^−1^ propidium iodide in water for 20 min. Sections were washed three times with water and mounted in wells of three-well Teflon microscope slides. A drop of 50% glycerol was added to each section and a cover slip was applied.

### Sample preparation for lignin analysis

Tissue samples were collected from inoculated and control plants at 2, 4, 6 and 8 DAI, excised, embedded, sectioned and mounted as described above. Sections were stained in a solution of 0.2% basic fuchsin dissolved in ClearSee, following a protocol described by Ursache, *et al*.^38^ except that the incubation time was reduced to 20 min, washed with water three times, stained with 0.1% calcofluor white in 20% EtOH for 10 min and washed with water three times. A drop of 50% glycerol was added to each section and a cover slip was applied.

### Imaging

To capture a depth of approximately 100 µm, images were obtained using the z-capture function of a Nikon A1R Laser Scanning Confocal microscope (Nikon, Tokyo, Japan) using two laser channels, DAPI (405 nm) and TRITC (561 nm). For each section, the laser power and gain settings were adjusted to avoid pixel saturation and minimise background. Images were processed with Nikon imaging software (NIS-Elements, www.microscope.healthcare.nikon.com/products/software/nis-elements) to obtain maximum-intensity projection images. IMARIS (version 9.2, imaris.oxinst.com) was then used to render 3D models of features (metaxylem vessels, feeding sites and nematodes) by creating surfaces from the fluorescence signal. In some cases, models were rotated to facilitate observation of details.

## Supplementary information

Supplementary Video S1.

Supplementary Video S2.

Supplementary information.

## Data Availability

All data, including image files, are available from the corresponding author on reasonable request.
