# Supplementary material for: Infection by cyst nematodes induces rapid remodelling of developing xylem vessels in wheat roots
Source: Sci Rep. 2020 Jun 3;10:9025. doi: 10.1038/s41598-020-66080-z (PMC7270153; doi:10.1038/s41598-020-66080-z)
Supplement: Supplementary file 3 — Supplementary information. [file 41598_2020_66080_MOESM3_ESM.docx]

**SUPPLEMENTARY DATA**

**Videos**

S1: Video of three-dimensional model from Fig. 1h showing further detailed rotation.

S2: Video of three-dimensional models from Fig. 2e-g showing 360° rotations.
